# Locally-applied 5-fluorouracil-loaded slow-release patch prevents pancreatic cancer growth in an orthotopic mouse model

**DOI:** 10.18632/oncotarget.17370

**Published:** 2017-04-22

**Authors:** In Kyong Shim, Hye-Jin Yi, Hee-Gyeong Yi, Chan Mi Lee, Yu Na Lee, Yeong-Jin Choi, Seong-Yun Jeong, Eunsung Jun, Robert M. Hoffman, Dong-Woo Cho, Song Cheol Kim

**Affiliations:** ^1^ Asan Institute for Life Science, University of Ulsan College of Medicine and Asan Medical Center, Seoul, South Korea; ^2^ Department of Mechanical Engineering, Pohang University of Science and Technology, Pohang, Kyungbuk, Korea; ^3^ Division of Integrative Biosciences and Biotechnology, Pohang University of Science and Technology, Pohang, Kyungbuk, Korea; ^4^ Department of Biomedical Sciences, University of Ulsan College of Medicine, Seoul, South Korea; ^5^ Department of Surgery, University of Ulsan College of Medicine & Asan Medical Center, Seoul, South Korea; ^6^ Department of Surgery, University of California, San Diego, CA, USA; ^7^ AntiCancer Inc., San Diego, CA, USA

**Keywords:** pancreatic cancer, orthotopic, nude mice, chemotherapy, patch

## Abstract

To obtain improved efficacy against pancreatic cancer, we investigated the efficacy and safety of a locally-applied 5-fluorouracil (5-FU)-loaded polymeric patch on pancreatic tumors in an orthotopic nude-mouse model. The 5-FU-releasing polymeric patch was produced by 3D printing. After application of the patch, it released the drug slowly for 4 weeks, and suppressed BxPC-3 pancreas cancer growth. Luciferase imaging of BxPC3-Luc cells implanted in the pancreas was performed longitudinally. The drug patch delivered a 30.2 times higher level of 5-FU than an intra-peritoneal (i.p.) bolus injection on day-1. High 5-FU levels were accumulated within one week by the patch. Four groups were compared for efficacy of 5-FU. Drug-free patch as a negative control (Group I); 30% 5-FU-loaded patch (4.8 mg) (Group II); 5-FU i.p. once (4.8 mg) (Group III); 5-FU i.p. once a week (1.2 mg), three times (Group IV). The tumor growth rate was significantly faster in Group I than Group II, III, IV (*p*=0.047 at day-8, *p*=0.022 at day-12, *p*=0.002 at day-18 and *p*=0.034 at day-21). All mice in Group III died of drug toxicity within two weeks after injection. Group II showed more effective suppression of tumor growth than Group IV (*p*=0.018 at day-12 and *p*=0.017 at day-21). Histological analysis showed extensive apoptosis in the TUNEL assay and by Ki -67 staining. Western blotting confirmed strong expression of cleaved caspase-3 in Group II. No significant changes were found hematologically and histologically in the liver, kidney and spleen in Groups I, II, IV but were found in Group III.

## INTRODUCTION

More than 70% of operated pancreatic-cancer patients develop local or metastatic recurrence after attempted curative resection. Adjuvant chemotherapy has been shown to be somewhat effective in recent clinical trials, increasing the low median-survival rate two-fold [1–5]. Improved therapy for this recalcitrant disease is urgently needed.

Since the amount of residual cancer cells is not large after pancreatic tumor resection, they can potentially be effectively eradicated with a small drug dose if it is properly delivered [6]. To improve adjuvant therapy of pancreatic cancer, we have designed a chemotherapeutic drug-loaded polymeric patch which can continuously administer small doses.

In a previous report, we described a three-dimensional printed (3DP) polymer patch loaded with a chemotherapy agent with controlled release and a manipulatable geometry which prevented tumor growth in a subcutaneous mouse model [6]. However, orthotopic models of pancreatic cancer are more patient-like since they are growing in a pancreatic micro-environment rather than a subcutaneous one [7–12].

In the present study, we investigated the efficacy and safety of the locally-applied chemotherapeutic-drug-loaded polymeric patch in an orthotopic murine pancreatic cancer model.

## RESULTS AND DISCUSSION

### Physical properties of the polymer patch

**Figure 1:**
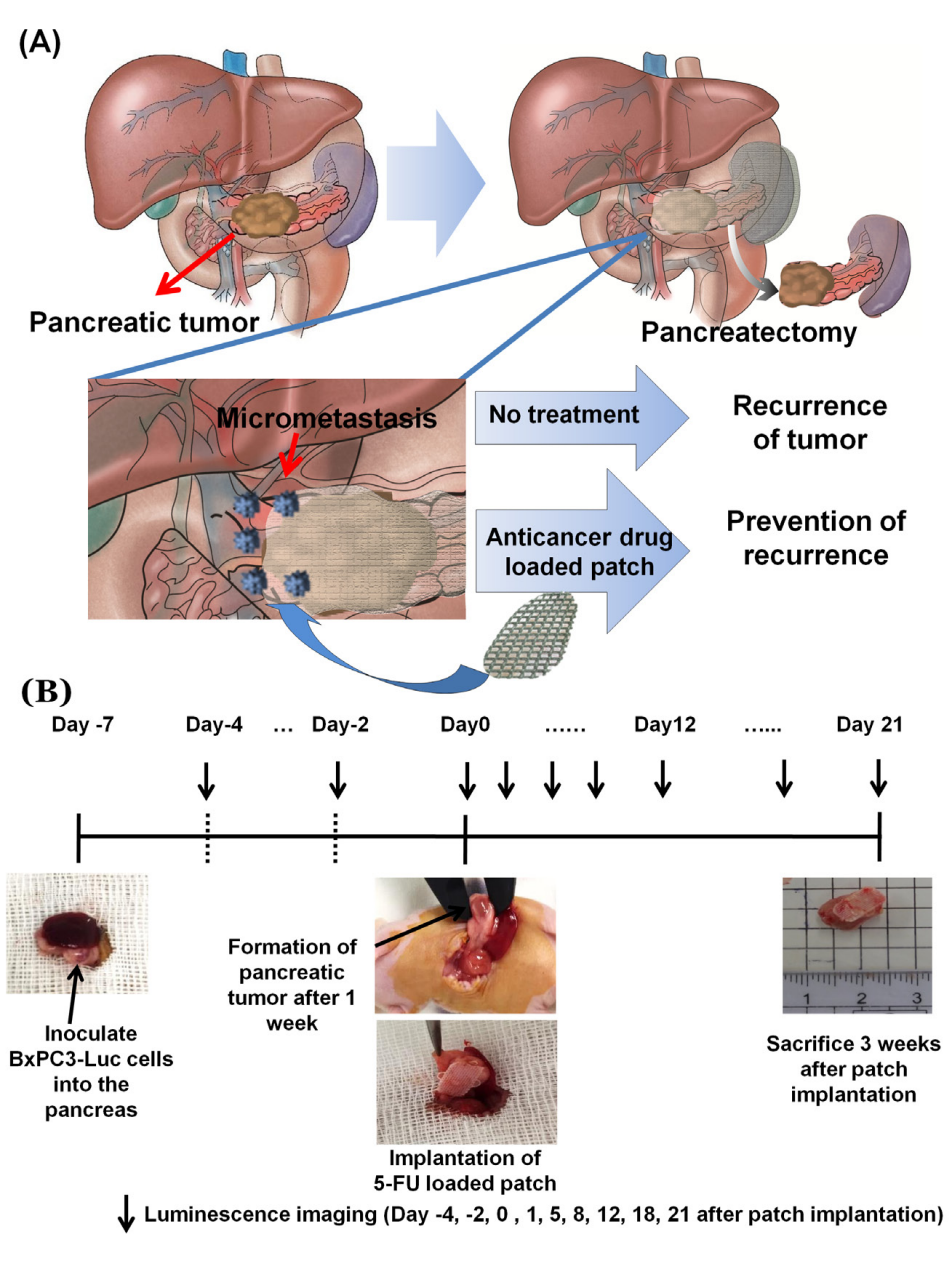
**A.** Schematic representation of the drug-loaded patch. **B.** Patch implantation process.

**Figure 2:**
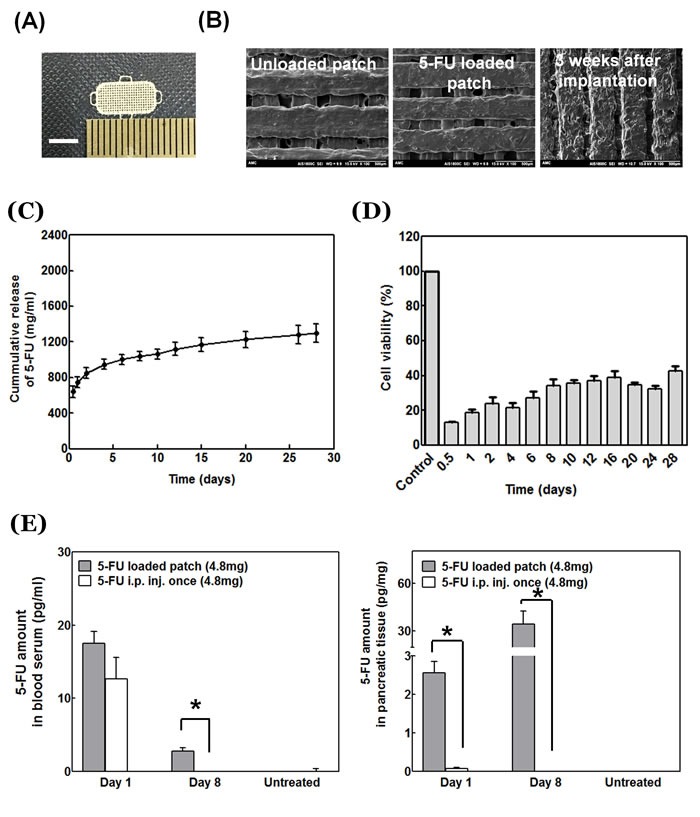
Physical properties of 5-FU-loaded patch **A.** Optical microscopy of the 5-FU-loaded patch. Scale bar: 5 mm. **B.** Scanning electron microscopy (Left: Unloaded patch; Middle: 5-FU-loaded patch; Right: 5-FU-loaded patch after implantation *in vivo* for three weeks. **C.** Cumulative release profile of 5-FU loaded patch. **D.** Cytotoxicity of 5-FU-loaded patch *in vitro*. **E.** Patch-delivered 5-FU concentration in mouse pancreas tissue and blood serum.

A schematic representation of a polymer patch and its implementation are shown in Figure 1A. The patch is described detailed in our previous publication [9]. The shape and size of the patch are adjustable. The patch has an average weight of 8 mg, a size of 10 × 5 mm. The patch has a loop at each corner such that it can cover a tumor which has a three-dimensional round shape. The patch can be fixed at the desired site with suture. The surface of the patch with or without 5-FU was imaged with scanning electron microscopy (SEM) (Figure 2).

*In vivo* degradation was analyzed by implanting the patch onto the orthotopic pancreatic tumor and analyzed by SEM from the resected tumor as shown in Figure 2B. When the patch surface was examined by SEM three weeks after implantation on the orthotopic pancreatic tumor, we confirmed that the drug had been released through the orifices in the patch (Figure 2).

### *In vitro* efficacy of the patch on BxPC-3 cells

The amount of 5-FU released from the patch *in vitro* was measured using UV detection to verify the drug release function of the patch (Figure 2C). The patches weighed approximately 8 mg and were loaded with 30% w/w of 5-FU which is equivalent to 2.4 mg of drug per patch. The in-vitro release pattern of 5-FU showed an early burst release of approximately 26% of the drug within the first day. The drug was slowly released after that. The amount of eluted drug from day 7 to 28 was 11-30 µg/ml (Figure 2C). BXPC-3 cell proliferation was inhibited more than 60% compared to untreated cells after four weeks (Figure 2D).

### *In vivo* delivery of 5-FU to the pancreas by the patch

5-FU was injected (4.8 mg) i.p. once a week and compared with 5-FU delivery in animals with the patch. The pancreas was removed on days 1 and 8 after drug treatment. The amount of 5-FU in the pancreas was measured by liquid chromatography tandem-mass spectrometry (LC-MS/MS). In animals with the 5-FU-loaded patch, the 5-FU level in the pancreas was 2.56 ± 0.29 pg/mg on day 1; and 34.42 ± 8.12mg on day 8. In animals with i.p. delivery of 5-FU, the amount of 5-FU in the pancreas was 0.08 ± 0.02 pg/mg on day 1; and 0.01 ± 0.00 pg/mg on day 8. The patch delivered a much larger amount of drug to the pancreas and pancreatic tumor site than i.p. delivery (Figure 2E) (*p* = 0.018 at day 1, *p* = 0.024 at day 8). The 5-FU level in the blood had no significant difference at day 1, between animals with patch implantation and animals with bolus injection. However, a small amount of 5-FU (0.01± 0.00) was detected in the blood of a patch-implanted mouse at day 8.

### Efficacy of the patch on tumor growth

**Figure 3:**
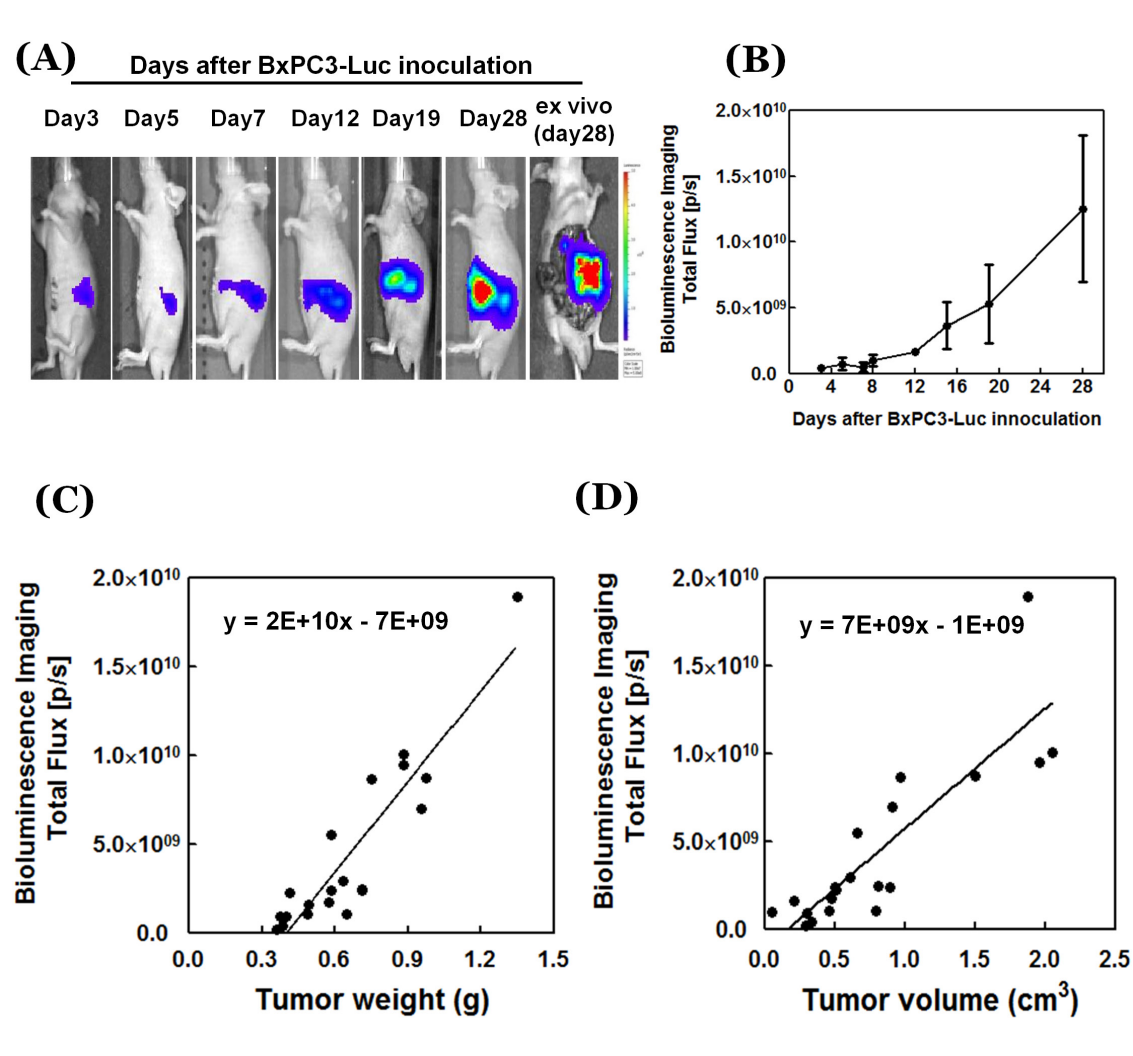
**A.** *In vivo* luciferase images of growing orthotopic pancreatic tumors (IVIS Spectrum) (see Figure D). **B.** Quantitative imaging data of pancreatic-cancer growth are presented as mean ± SD. **C.** Correlation between tumor weight photon flux and **D.** tumor volume photon flux. Please see Materials and Methods for details. Data (*n* = 20) are presented as linear regression and Pearson correlation by GraphPad Prism. P/S= photon/second.

In untreated mice and mice implanted with a drug-free patch, the orthotopic pancreatic cancer grew rapidly as shown by non-invasive imaging (Figure 3). The Pearson -correlation coefficient between tumor weight and image-calculated volume was 0.914, and correlation between actual tumor volume and image-calculated volume was 0.857. These results show that tumor-volume calculations obtained from the tumor image can reflect the actual tumor volume and weight.

**Figure 4:**
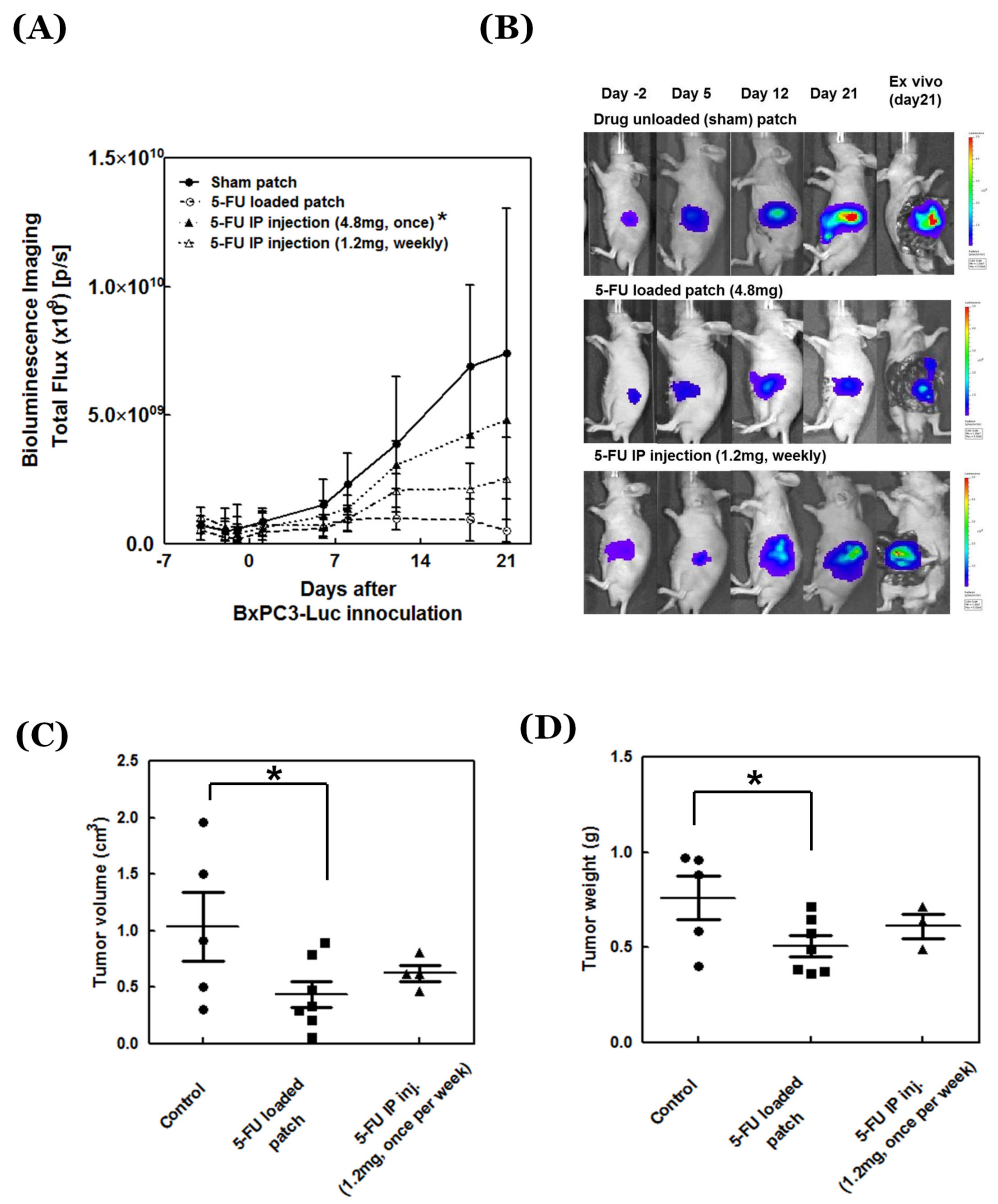
Antitumor efficacy of 5-FU-release patch **A.** Tumor growth curves of treated and untreated mice (*n* = 6). *The mouse number changed in the 5-FU i.p. injection group with high dose (4.8mg) due to mouse death (*n* = 6 at day -4, -2, -1, 1, 6, 8; *n* = 2 at day 12; and *n* = 1 at day 18, 21). **B.** Representative images of the treated and untreated tumors. **C.** Tumor weight and **D.** tumor volume at day 21 after drug treatment.

In mice treated with i.p. 5-FU, non-invasive imaging showed continuous tumor-growth suppression but it was not significantly different from the untreated control (*p* = 0.132 at day 8, 0.057 at day 18 and 0.216 at day 21). When 4.8 mg 5-FU was injected i.p. once, the growth of the pancreatic cancer was suppressed at the beginning. However, most of the mice died after two weeks, and the pancreatic cancer of one surviving mouse showed increased tumor growth after two weeks. The strongest tumor-suppression efficacy was with the patch implantation compared to animals implanted with an unloaded patch (Figure 4) (*p* = 0.031 at day 8, 0.002 at day 18 and 0.015 at day 21).

At the end of the treatment period on day 21, tumors were resected from mice in each group and tumor volume and weight were directly measured. Both tumor weight and volume were significantly inhibited by the drug-loaded patch (*p* = 0.029 for volume and *p* = 0.035 for weight). 5-FU delivered i.p. showed an inhibitory trend for tumor volume and weight but there is no significant difference (Figure 4D, 4E) for tumor volume (*p* = 0.434) or for tumor weight (*p* = 0.891).

### Immunohistochemical staining of the treated pancreatic tumor

**Figure 5:**
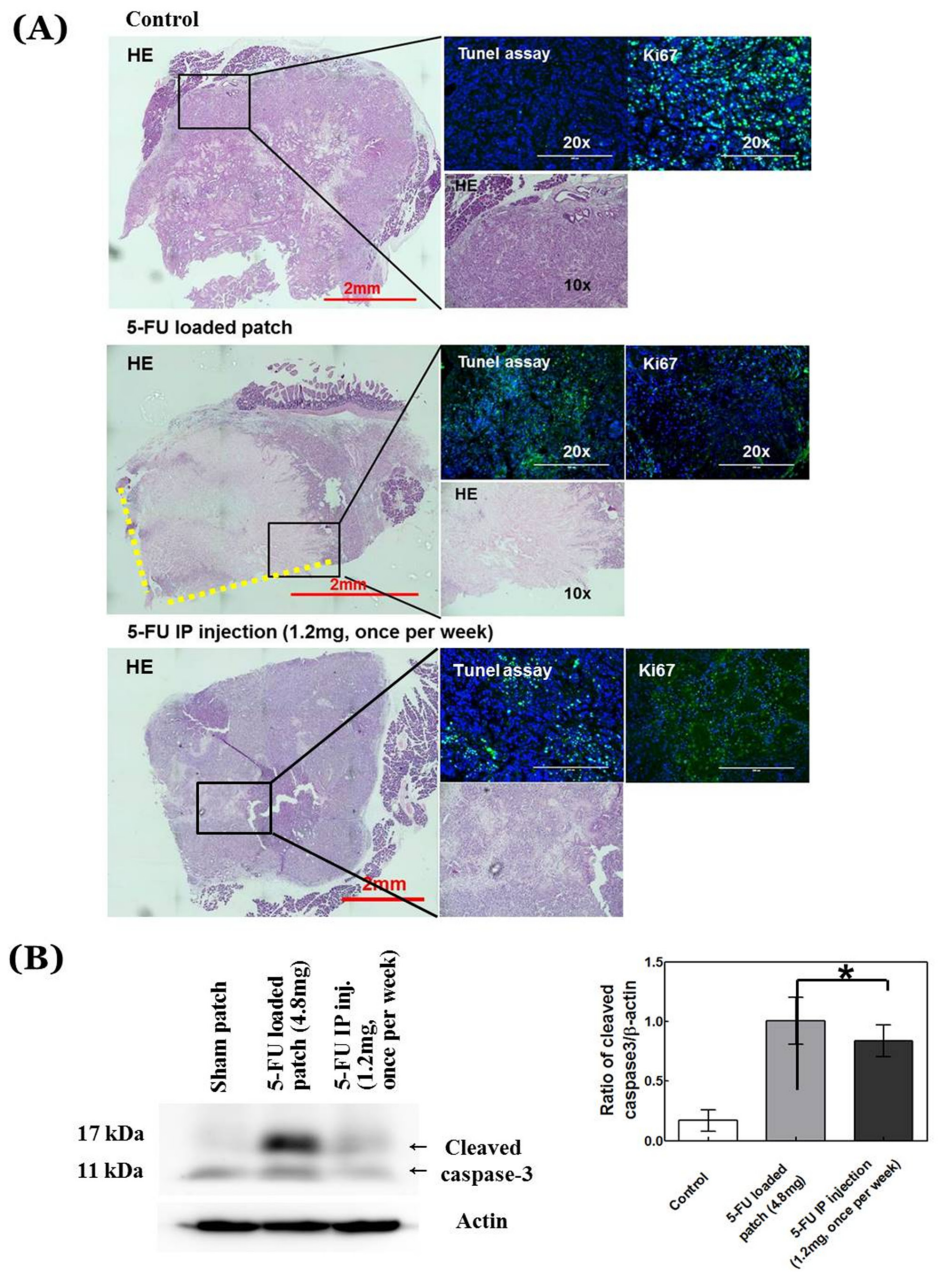
Histological analysis **A.** Images of hematoxylin and eosin (H&E)-stained pancreatic-cancer tissue sections from treated and untreated mice along with TUNEL and KI-67 staining for each condition. **B.** Cleaved caspase-3 from treated and untreated mice. H&E scale bar, 2 mm; fluorescence-image scale bar, 200 µm. Yellow-dotted line shows contact side of 5-FU-loaded patch.

Most of the cells were stained with KI-67 and a few of them stained with TUNEL assay in the tumors covered with the sham patch (Figure 5A). In contrast, most cells around the adhesive face of the patch were stained with TUNEL in mice treated with the 5-FU-loaded patch and they did not stain with KI-67. However, some parts of the pancreatic cancer showed growth on the part of the tumor where the patch could not reach. Western blotting showed strong expression of cleaved caspase-3 indicating more apoptosis in the patch group than the control group (*p* = 0.018) (Figure 5). The bolus-injection group also showed a similar pattern as the mice with the drug-loaded patch. However, in the bolus-treated mice, cells inside the tumor were stained with KI-67 (Figure 5B).

### Survival and hematological and histological toxicity

**Figure 6:**
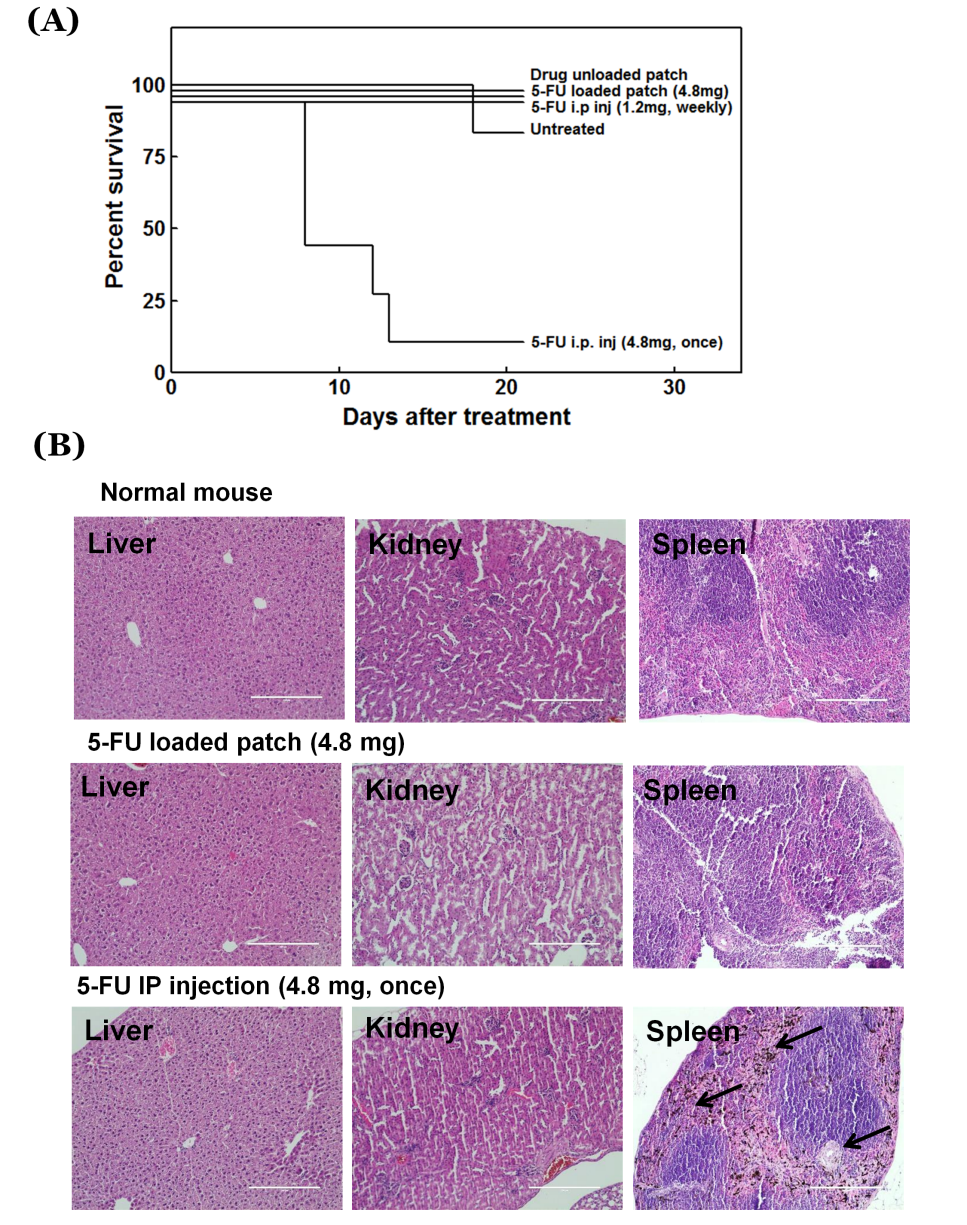
**A.** Survival rate of treated and untreated mice. **B.** H&E-stained liver (left panel), kidney (middle panel), and spleen (right panel) sections. Black arrow: pigment. Scale bar: 200 µm.

The experiment period was 28 days after cancer cell inoculation, which is not sufficient for metastasis to occur or to cause mouse death as a result of tumor growth. All mice treated with the sham patch, 5-FU-loaded patch and 5-FU bolus injection with low dose survived until the end of the experimental period. However, for the mice treated with high-dose bolus 5-FU all died, except one, during the experimental period due to drug overdose (Figure 6A).

All groups treated with 5-FU (patch and i.p.) showed a slight decrease in hematological values. However, in the high-dose (i.p.) group, most hematological factors such as WBC, RBC, HGB, HCT, etc., decreased more than 50%, especially reticulocytes, which showed a decrease of more than 90% (Table 1). Histologically normal organs showed no difference between mice treated with the drug-loaded patch and untreated animals. However, the spleen from a mouse injected with high dose 5-FU treated mouse showed loss of lymphoid tissue, fibrosis, and accumulation of iron pigment (Figure 6).

**Table 1:** Hematologic parameters from mice with various treatments for one week

Hematology	Unit	Control	5-FU patch	5-FU IP inj. (4.8mg, once)	5-FU IP inj. (1.2mg, once/week)
		Ave±SD	Ave±SD	Ave±SD	Ave±SD
WBC	10e3/uL	7.12±2.01	5.18±0.58^b^	1.95±0.75^b^	4.68±0.31^b^
RBC	10e6/uL	10.00±0.28	8.86±0.14	4.49±0.64^b^	8.09±0.66
HGB	g/dL	14.90±0.36	13.87±0.23	6.53±0.90^b^	12.57±1.08
HCT	%	48.83±1.25	47.30±1.08	22.73±3.09^b^	43.57±2.65
PLT	10e3/uL	1259±58	1179.±454	1019±187	950±780
Neut	10e3/uL	0.53±0.22	0.62±0.16	0.04±0.01^b^	0.29±0.08^b^
Lymph	10e3/uL	6.27±1.75	4.28±0.33	1.88±0.73^b^	4.19±0.41
Mono	10e3/uL	0.07±0.02	0.07±0.03	0.00±0.00	0.04±0.01
Luc	10e3/uL	0.08±0.05	0.02±0.01	0.01±0.01^b^	0.04±0.03
%Retic	%	3.56±0.09	9.49±1.40^a^	0.34±0.19^b^	18.38±14.37^a^
#Retic	10e9/L	356.23±14.87	839±112^a^	14.50±6.00^b^	1446±1061^a^

Data presented as mean±SD, *n* = 4. ^a^ and ^b^ represent significant increase and decrease at *p* < 0.05 compared to control values, respectively.

There was 33.5 times more 5-FU delivered to the pancreas with the patch than with a bolus i.p. 5-FU injection. With the patch, high levels of 5-FU remained in the pancreas even after one week, and was 21.9 times higher than bolus injection.

The orthotopic pancreatic cancer model is more appropriate for the patch than subcutaneous models, as the orthotopic mouse patch can be readily translated to the clinic.

Previously-developed concepts and strategies of highly-selective tumor targeting can take advantage of molecular targeting of tumors, including tissue-selective therapy which focuses on unique differences between normal and tumor tissues [14–19].

## CONCLUSION

The results of the present study show that a polymer patch constructed with 3D printing can release 5-FU for more than four weeks after attachment to the pancreas. Since the patch is directly attached to the organ, the 5-FU concentration in the blood is negligible but very high in the pancreatic tumor. The growth of pancreatic cancer was arrested by the patch with minimal toxicity. 3DP techniques have been previously demonstrated to produce implants that have precisely defined, micro- and macro architectures which can be applied effectively for complex release of multiple types of drugs [20–25]. In the clinic, the patch can be used after pancreatic cancer resection to prevent recurrence.

## MATERIALS AND METHODS

### 5-FU-loaded polymer patch

A detailed description of patch production procedures can be found in our previous publication [6]. A polymer solution was prepared by melting polycaprolactone (PCL) (Polysciences, Inc. PA, USA) and poly(lactide-co-glycolide) (PLGA) (lactide: glycolide = 85:15, Sigma-Aldrich, MO, USA) at a ratio of 1:1 at 140°C. 5-FU powder was mixed with the polymer solution at a 3:7 w/w ratio and loaded into the reservoir of a 3D printer. The mixture solution was extruded from the reservoir at 600 kPa in the printing head of an in-house-manufactured extrusion-based 3D printer with a multi-head deposition system (MHDS) [10].

### Release profile of the 5-FU-loaded patch

The concentration of 5-FU released from the patch in PBS was determined by measuring the absorbance at λ = 265 nm using a UV-Vis spectrophotometer (Spectramax 190, molecular device, CA USA).

### Efficacy of 5-FU-releasing solution *in vitro*

BxPC3-Luc cells (3×10^3^) were seeded into 96-well plates for 24 hours prior to treatment (*n* = 4). 5-FU-releasing medium or fresh complete culture medium was added to the wells. After 48 hours treatment with drug-releasing solution, cell viability was assessed using the Cell Counting Kit-8 (CCK-8) assay (Dojindo Laboratories, Kumamoto, Japan). The absorbance was measured using a microplate reader at 450 nm (Sunrise, TECAN, Switzerland). Cytotoxicity relative to control was determined.

### Quantitation of 5-FU-released in the pancreas after patch implantation

5-FU-loaded (30%) patches in the pancreas of BALC/c nude mice were attached to the pancreatic tumors 7-days after inoculation of BxPC3-Luc cells. 5-FU (1.2 mg), dissolved in PBS, was injected on the same day, and 1.2 mg of the drug was once again injected a week after the first injection for comparison with the patch. The pancreas and serum were collected on the first day of patch implantation or drug treatment and on day 8 of the study as well.

Analysis of 5-FU was performed with a liquid chromatography-tandem mass spectrometry system (LC-MS/MS). Glacial acetic acid (5 μl), ethyl acetate (500 μl), and internal standard solution (0.001 mg/ml 5-bromouracil) (50 μl) were added to serum (50 μl) or tissue (50 mg). For tissue, the sample was homogenized using a TissueLyzer (Qiagen, Germany) and the supernatant was collected after centrifugation. For serum, the solution was mixed well for 5 min and centrifuged at 10,000 rpm for 5 min (Centrifuge 5415R, Eppendorf, Germany). The supernatant was transferred to a new tube. The supernatant was dried under vacuum, then stored at -20°C until analysis. The dried material was reconstituted with acetonitrile (20 μl)/water/formic acid (97/3/0.1, v/v/v) before LC-MS/MS analysis. A LC-MS/MS system equipped with a 1290 HPLC (Agilent), Qtrap 5500 (ABSciex) and hydrophilic interaction column (Shodex Asahipak NH2P-50 2D column (5 μm, 150 × 2 mm), Phenomenex, USA) was used. The separation gradient for 5-FU analysis used a mobile phase A (0.1% formic acid in water) and mobile phase B (0.1% formic acid in acetonitrile) and proceeded at 300 µl/min at 23°C. The separation gradient was as follows: hold at 3-40% A for 3 min; 40 to 50% A for 1.6 min; 50 to 80% A for 1.4 min; hold at 80% A for 8 min; 80 to 0% A for 1 min; 0 to 3% A for 2 min and then hold at 3% A for 3 min. The multiple-reaction-monitoring (MRM) mode was used in negative-ion mode, and the extracted-ion chromatogram, corresponding to the specific transition (128.8/42.1 for 5-FU; 188.7/42.1 for 5-bromouracil), was used for quantification. The calibration range for 5-FU was 0.0001−10 ng/ml (r2 ≥ 0.99). The background level of the untreated mice was used as a normalization control.

### Cell line

The BxPC3-Luc human pancreatic adenocarcinoma cell line, transduced with luciferase, was cultured in RPMI-1640 (RPMI; GIBCO) containing 10 % heat-inactivation fetal bovine serum (FBS; GIBCO, MD, USA) and 1 % Antibiotic-Antimycotic (AA; GIBCO, MD, USA) in a humidified incubator at 37°C with 5% CO_2_ atmosphere. BxPC3-Luc cells were selected in multiple steps and a clone was selected for high luciferase intensity.

### Mice

To prepare the xenograft mouse tumor model, BALB/c nude mice (Orient Bio Inc., Sungnam, Korea) were used (male, 6 weeks). All mouse experiments within the guidelines of the protocol were reviewed by the Institutional Animal Care and Use Committee of Asan Institute for Life Science [11]. BxPC-3-Luc cells were harvested (1×10^6^) and resuspended in serum-free RPMI-1640 (10 µl) medium mixed with Matrigel (2:3 ratio). After inhalational anesthesia with isoflurane, the entire pancreatic body and spleen were exposed to the outside of the peritoneal cavity using a blunt forceps, 10 µl cells (1×10^6^) in a Matrigel suspension were injected slowly using a 29 gauge syringe into the tail of the pancreas. The needle was removed from the pancreas and after a wait of 1-2 minutes, mice were inspected for hemorrhage and leakage. The pancreas and spleen were carefully repositioned back to the peritoneal cavity and the abdominal wall was closed with a 6.0 vicryl suture and abdominal skin closed using 5.0 black silk sutures [25].

### Implantation of 5-FU-loaded patch

Two patches were fixed on the front and the back of the pancreas so that the drug could be delivered three-dimensionally (Figure 1B). Mice were randomly divided into 4 groups at day 7 after orthotopic cancer-cell inoculation; Group I: implanted with a drug-free patch (sham patch); Group II: implanted with a 30% 5-FU-drug-loaded patch (4.8 mg); Group III: 5-FU i.p. injection once (4.8 mg); and Group IV: 5-FU i.p. injection once a week (1.2 mg), three times. All experimental groups started with *n* = 6 to 8 mice at the time of treatment initiation. Patches were surgically fixed on the pancreatic tumor. For i.p. injection, the 5-FU was dissolved in PBS and slowly injected on day 7. In group III, 4.8 mg of 5-FU was injected into the abdominal cavity once. In group IV, 1.2 mg of 5-FU dissolved in PBS was injected once a week (day 0, 7, 14).

### Optical imaging of the orthotopic pancreatic tumor

Mice were intraperitoneally injected with D-luciferin (0.3 mg; Perkin Elmer Inc.). Whole-body luminescence imaging with an IVIS Spectrum (Caliper Inc., Alameda, CA) was performed every 3 minutes until radiance values reached the maximum. The region of the interested (ROI) level was measured with the radiance (photons/s/cm^2^/sr) using an analysis program, Living Image 4.4 (Caliper Life Sciences, PerkinElmer Inc.).

### Tumor measurement

The volume of the tumor was measured with calipers after removal at the end of the experiment. Tumor volume [mm^3^] = π/6 × (L) × (W) × (D). Tumors were also weighed. Tumor weight or volume were correlated to image size.

### Immunohistochemical staining

After sacrificing the mice at day 21, the tumors were removed and fixed in 4% neutral-buffered para-formaldehyde and embedded in paraffin. Paraffin blocks were cut into 4 µm sections and were reviewed histologically after hematoxylin and eosin staining. Paraffin sections were deparaffinized and then rehydrated. After microwave antigen retrieval, non-specific binding sites were blocked with PBS containing 10% normal goat serum. The sections were further incubated with the primary antibodies against Ki-67 (Dako, Glostrup, Denmark) followed by an Alexa 488-conjugated secondary antibody (Thermo Fisher Scientific, Danvers, Massachusetts, USA). TUNEL staining was performed using *in situ* cell-death detection kit (Roche Diagnostics GmbH, Mannheim, Germany) following the manufacturer's protocol. The samples were mounted using Prolong Gold antifade mountant with DAPI (Thermo Fisher Scientific). Images were obtained using an EVOS-fluorescence microscope (Thermo Fisher Scientific).

### Western blotting

Protein samples were extracted from frozen tumors removed from sacrificed mice at day 21. For whole-lysate extraction, RIPA Lysis and Extraction Buffer (Biosensing, Seongnam, Republic of Korea) supplemented with a protease-inhibitor cocktail (Complete Mini, ethylenediaminetetraacetic acid-free; [Roche Diagnostics]) was used. The protein concentration of each sample was determined using a BCA protein assay kit (Thermo Fisher Scientific). Protein samples were resolved on a 10% polyacrylamide gel and were transferred onto nitrocellulose membranes (Trans-Blot^®^ Turbo™ nitrocellulose; Bio-Rad, Hercules, CA). The membranes were blocked using 5% nonfat dry milk (AppliChem, Cheshire, CT, USA) in Tris-buffered saline Tween-20 (TBST; pH 7.6; 20 mM Tris-HCl, 150 mM NaCl and 0.1 % Tween 20) at room temperature (24°C) for 30 min and were incubated overnight at 4°C with cleaved caspase-3 rabbit monoclonal antibody (1:1000 dilution, Cell Signaling Technology, Danvers, MA, USA). The membranes were then rinsed three times with TBST (pH 7.6) for 10 min at room temperature and incubated with horseradish peroxidase (HRP)-conjugated HRP-conjugated goat anti-rabbit IgG (1:10000 dilution) (Santa Cruz Biotechnology, Santa Cruz, CA, USA) at room temperature for 1 hr with gentle agitation. Membranes were washed five times with TBST (pH 7.6) for 30 min at room temperature. Protein signals were detected using an enhanced chemiluminescence (ECL) solution (SuperSignal™ West Femto; Thermo Fisher Scientific). Images were captured using an ImageQuant LAS 4000 system (GE Healthcare Biosciences, Piscataway, NJ, USA). The blots were stripped using Restore™ PLUS (Thermo Fisher Scientific) stripping buffer and were reprobed for β-actin (internal control) using the same methods. Mouse monoclonal anti-human actin antibody (1:20000 dilution, Calbiochem, Darmstadt, Germany) was used as the primary antibody, and HRP-conjugated goat anti-mouse antibody (1:1000 dilution, Santa Cruz Biotechnology) was used as the secondary antibody.

### *In vivo* toxicity

BALB/c mice (7 weeks) were given an intraperitoneal injection of 4.8 mg 5-FU once or 1.2 mg 5-FU once per week. Also, mice were treated by transplantation of two 5-FU-loaded patches (4.8 mg) or transplantation of sham patches. Mice were sacrificed on day 7 and samples of blood were taken from the abdominal vein. This experiment was performed in triplicate. All external features and orifices were visually examined and lesions which showed any abnormal morphology were recorded. Blood samples were collected for hematology determinations in tubes with EDTA-2 K as an anticoagulant. Hematology determinations included white-blood-cell (WBCs) count, red-blood-cell (RBC) count, hemoglobin concentration, hematocrit content, platelet count, differential leucocyte count (neutrophils, lymphocytes, monocytes), reticulocyte count, using an Advia 120 Hematology analyzer (Bayer Healthcare, Myerstown, PA, USA). The liver, spleen, and kidney, were analyzed for toxicity histologically. All organs/tissues were fixed in 10% neutral-buffered formalin and were processed and trimmed, embedded in paraffin, sectioned at a thickness of 4-6 µm, and stained with hematoxylin and eosin for microscopic examination.

### Statistical analysis

Statistical significance was determined using GraphPad Prism 5 (GraphPad Software, San Diego, CA, USA) for tumor analysis. Data were analyzed by Pearson correlation for comparison of image size and tumor weight or volume. Survival among the groups was analyzed using the Log-Rank test for trend and pair-wise comparisons. The statistical significance of the differences between groups was analyzed with the Student's *t*-test and a two-way analysis of variance. *P* < 0.05 indicates a statistically-significant difference. The data are presented as the mean ± standard deviation, with the number of samples indicated in the figure legends.

## References

[1] Oettle H, Post S, Neuhaus P, Gellert K, Langrehr J, Ridwelski K, Schramm H, Fahlke J, Zuelke C, Burkart C, Gutberlet K, Kettner E, Schmalenberg H (2007). Adjuvant chemotherapy with gemcitabine vs observation in patients undergoing curative-intent resection of pancreatic cancer: a randomized controlled trial. JAMA.

[2] Ueno H, Kosuge T, Matsuyama Y, Yamamoto J, Nakao A, Egawa S, Doi R, Monden M, Hatori T, Tanaka M, Shimada M, Kanemitsu K (2009). A randomised phase III trial comparing gemcitabine with surgery-only in patients with resected pancreatic cancer: Japanese Study Group of Adjuvant Therapy for Pancreatic Cancer. Br J Cancer.

[3] Regine WF, Winter KA, Abrams RA, Safran H, Hoffman JP, Konski A, Benson AB, Macdonald JS, Kudrimoti MR, Fromm ML, Haddock MG, Schaefer P, Willett CG (2008). Fluorouracil vs gemcitabine chemotherapy before and after fluorouracil-based chemoradiation following resection of pancreatic adenocarcinoma: a randomized controlled trial. JAMA.

[4] Neoptolemos JP, Stocken DD, Tudur Smith C, Bassi C, Ghaneh P, Owen E, Moore M, Padbury R, Doi R, Smith D, Buchler MW (2009). Adjuvant 5-fluorouracil and folinic acid vs observation for pancreatic cancer: composite data from the ESPAC-1 and -3(v1) trials. Br J Cancer.

[5] Neoptolemos JP, Stocken DD, Bassi C, Ghaneh P, Cunningham D, Goldstein D, Padbury R, Moore MJ, Gallinger S, Mariette C, Wente MN, Izbicki JR, Friess H (2010). Adjuvant chemotherapy with fluorouracil plus folinic acid vs gemcitabine following pancreatic cancer resection: a randomized controlled trial. JAMA.

[6] Yi HG, Choi YJ, Kang KS, Hong JM, Pati RG, Park MN, Shim IK, Lee CM, Kim SC, Cho DW (2016). A 3D-printed local drug delivery patch for pancreatic cancer growth suppression. J Control Release.

[7] Fu X, Guadagni F, Hoffman RM (1992). A metastatic nude-mouse model of human pancreatic cancer constructed orthotopically from histologically intact patient specimens. Proc Natl Acad Sci USA.

[8] Furukawa T, Kubota T, Watanabe M, Kitajima M, Hoffman RM (1993). A novel “patient-like” treatment model of human pancreatic cancer constructed using orthotopic transplantation of histologically intact human tumor tissue in nude mice. Cancer Res.

[9] Bouvet M, Wang JW, Nardin SR, Nassirpour R, Yang M, Baranov E, Jiang P, Moossa AR, Hoffman RM (2002). Real-time optical imaging of primary tumor growth and multiple metastatic events in a pancreatic cancer orthotopic model. Cancer Res.

[10] Hoffman RM (2015). Patient-derived orthotopic xenografts: better mimic of metastasis than subcutaneous xenografts. Nature Reviews Cancer.

[11] Hoffman RM Orthotopic metastatic mouse models for anticancer drug discovery and evaluation: a bridge to the clinic. Investigational New Drugs.

[12] Hoffman RM (1994). Orthotopic is orthodox: Why are orthotopic-transplant metastatic models different from all other models?. J Cellular Biochem.

[13] Lee JS, Cha HD, Shim JH, Jung JW, Kim JY, Cho DW (2012). Effect of pore architecture and stacking direction on mechanical properties of solid freeform fabrication-based scaffold for bone tissue engineering. J Biomed Mater Res A.

[14] Blagosklonny MV (2003). Matching targets for selective cancer therapy. Drug Discov Today.

[15] Blagosklonny MV (2005). Teratogens as anti-cancer drugs. Cell Cycle.

[16] Blagosklonny MV (2001). Treatment with inhibitors of caspases, that are substrates of drug ransporters, selectively permits chemotherapy-induced apoptosis in multidrug-resistant cells but protects normal cells. Leukemia.

[17] Blagosklonny MV (2006). Target for cancer therapy: proliferating cells or stem cells. Leukemia.

[18] Apontes P, Leontieva OV, Demidenko ZN, Li F, Blagosklonny MV (2011). Exploring long-term protection of normal human fibroblasts and epithelial cells from chemotherapy in cell culture. Oncotarget.

[19] Blagosklonny MV (2003). Tissue-selective therapy of cancer. Br J Cancer.

[20] Yi HG, Choi YJ, Kang KS, Hong JM, Pati RG, Park MN, Shim IK, Lee CM, Kim SC, Cho DW (2016). A 3D-printed local drug delivery patch for pancreatic cancer growth suppression. J Control Release.

[21] Ursan ID, Chiu L, Pierce A (2013). Three-dimensional drug printing: a structured review. J Am Pharm Assoc.

[22] Yu DG, Zhu LM, Branford-White CJ, Yang XL (2008). Three-dimensional printing in pharmaceutics: promises and problems. J Pharm Sci.

[23] Lee JS, Cha HD, Shim JH, Jung JW, Kim JY, Cho DW (2012). Effect of pore architecture and stacking direction on mechanical properties of solid freeform fabrication-based scaffold for bone tissue engineering. J Biomed Mater Res A.

[24] Kim JY, Yoon JJ, Park EK, Kim DS, Kim SY, Cho DW (2009). Cell adhesion and proliferation evaluation of SFF-based biodegradable scaffolds fabricated using a multi-head deposition system. Biofabrication.

[25] Song SY, Kim KP, Jeong SY, Park J, Park J, Jung J, Chung HK, Lee SW, Seo MH, Lee JS, Jung KH, Choi EK (2016). Polymeric nanoparticle-docetaxel for the treatment of advanced solid tumors: phase I clinical trial and preclinical data from an orthotopic pancreatic cancer model. Oncotarget.

